# Cardiac tissue-derived extracellular matrix scaffolds for myocardial repair: advantages and challenges

**DOI:** 10.1093/rb/rbz017

**Published:** 2019-04-22

**Authors:** Pawan KC, Yi Hong, Ge Zhang

**Affiliations:** 1 Department of Biomedical Engineering, The University of Akron, Olson Research Center, Room 301L, 260 S Forge Street, Akron, OH, USA; 2 Department of Bioengineering, University of Texas at Arlington, 500 UTA Blvd, Room 240, Arlington, TX, USA

**Keywords:** myocardial infarction, decellularized extracellular matrix, scaffold, cardiac tissue engineering

## Abstract

Decellularized extracellular matrix (dECM) derived from myocardium has been widely explored as a nature scaffold for cardiac tissue engineering applications. Cardiac dECM offers many unique advantages such as preservation of organ-specific ECM microstructure and composition, demonstration of tissue-mimetic mechanical properties and retention of biochemical cues in favor of subsequent recellularization. However, current processes of dECM decellularization and recellularization still face many challenges including the need for balance between cell removal and extracellular matrix preservation, efficient recellularization of dECM for obtaining homogenous cell distribution, tailoring material properties of dECM for enhancing bioactivity and prevascularization of thick dECM. This review summarizes the recent progresses of using dECM scaffold for cardiac repair and discusses its major advantages and challenges for producing biomimetic cardiac patch.

## Milestones of cardiac decellularized extracellular matrix research

The decellularization strategies and their applications in regenerative medicine have been gradually explored since late 1940s. The first pioneer study of tissue decellularization with quantitative measurement was conducted by William E. Poel in 1948 [1]. In this study, acellular homogenate was generated from muscles through complete pulverization of tissue at –70°C by pounding followed by homogenization of pulverized and thawed tissue sample in water using a cylinder and a closely fitting rotating plunger [2]. Following this work, decellularization strategies have been used on tissue biopsies for isolation of tissue-specific extracellular matrix (ECM) using various approaches including chemical treatments (e.g. acids and bases, detergents, alcohols), biological treatments (e.g. enzymes, chelating agents) and physical treatments (e.g. pressure, mechanical, freezing and thawing and electroporation) [3–7]. In 1995, decellularization research entered into another phase of development when Badylak *et al.* reported the application of using decellularized porcine small intestinal submucosa (SIS) graft for Achilles tendon repair. When implanted in a dog model with an Achilles tendon defect, acellular porcine SIS graft has shown to accelerate the wound healing by forming new connective tissues [8]. Subsequently, porcine SIS graft has been explored to treat many other injured tissues such as abdomen, skin, trachea, cornea and myocardium [9–13]. The promising results from these studies led to numerous investigations to assess the feasibility of using tissue-derived ECM after decellularization as a natural scaffold for tissue repair and regeneration.

**Figure 1.**
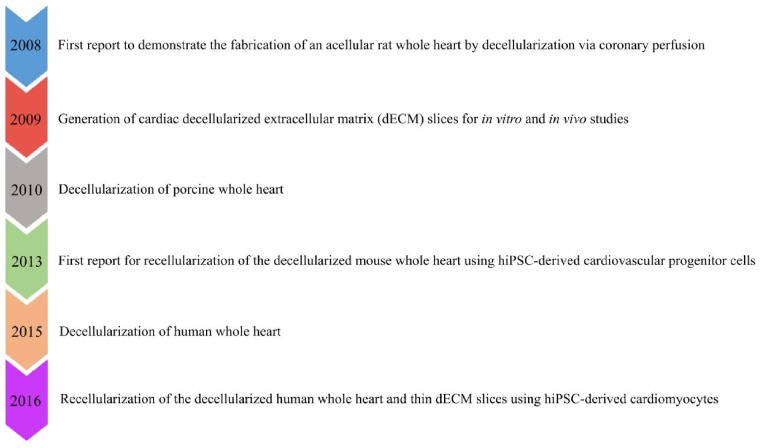
Timeline of major milestones using dECM scaffold for myocardial repair. hiPSC, human induced pluripotent stem cell; dECM, decellularized extracellular matrix

The application of decellularization in cardiac tissue engineering has rapidly progressed in the past 10 years (Fig. 1). In 2008, Ott *et al.* first reported the development of an acellular rat whole heart (WH) via coronary perfusion using the solution containing 1% sodium dodecyl sulfate (SDS) and 1% Triton X-100 in deionized water [14]. The decellularized rat WH preserved the complex ECM composition as well as retained intact chamber geometry and perfusable vascular architecture. When reseeded the acellular rat WH with cardiac and endothelial cells using a perfusion bioreactor culture system, the recellularized WH resulted macroscopic contractions, leading to generate 2% of adult and 25% of 16-week fetal heart physiological functions [14]. In the subsequent years, whole-organ decellularization approaches have been extended to the larger hearts from porcine and human origin to realize human-size functional cardiac grafts [15–24]. For example, Wainwright *et al.* conducted the first study to generate acellular porcine WH via a retrograde coronary perfusion using successive treatments with 0.02% trypsin/0.05% EDTA/0.05% sodium azide solution, 3% Triton X-100/0.05% EDTA/0.05% sodium azide solution, followed by 4% deoxycholic acid solution. The decellularized porcine heart preserved ECM composition (e.g. collagen, elastin and glycosaminoglycans), retained mechanical integrity and supported cardiac cells *in vitro* [25]. Compared to porcine heart, decellularization method has been applied to a limited number of human WH to obtain dECM scaffold. Sanchez *et al.* produced first acellular human WH scaffold by perfusion decellularization using a detergent solution containing 1% SDS in deionized water. After decellularization, the human WH preserved the three-dimensional (3D) architecture, chamber geometry, vascularity and mechanical anisotropy. When reseeded with parenchymal and vascular cells, the human WH promoted cardiocyte gene expression and electrical coupling [24]. In addition to produce acellular organ, decellularization has also been exploited to derive cardiac dECM slices. Various groups have fabricated cardiac dECM slices through decellularization using native cardiac tissues from multiple species including rat, mouse, pig and human [26–29]. Moreover, the acellular WHs or cardiac dECM slices have been explored for many *in vitro* and *in vivo* studies to thoroughly investigate the dECM properties and cell–matrix interaction (e.g. cell adhesion, proliferation and differentiation) using various cell types including mesenchymal stem cells (MSCs), embryonic stem cells (ESCs) and induced pluripotent stem cells (iPSCs) [30–34]. For instance, Lu and Lin *et al.* reported for the first time of seeding human iPSC-derived cardiovascular progenitor cells on decellularized mouse WH via perfusion through the cannula that was connected to the aorta. After seeding, the recellularized WH promoted differentiation and maturation of iPSC-derived cardiovascular progenitor cells toward cardiomyocytes (CMs), smooth muscle cells and endothelial cells that resulted the engineered myocardium with vessel-like structures, spontaneous contraction, intracellular Ca^2+^ transients and drug response [35]. Similarly, Guyette *et al.* recently reported the repopulation of decellularized human WH and decellularized human myocardial slices (200 µm thick) with human iPSC-derived CMs. When grown under biomimetic culture conditions, the seeded dECM scaffolds developed force-generating myocardial tissue with spontaneous contraction and showed electrical conductivity as well as metabolic function [36]. These studies have proved the great potential of using cardiac decellularized ECM as a natural platform for cardiac tissue engineering applications.

## Advantages of cardiac decellularized extracellular matrix

**Figure 2.**
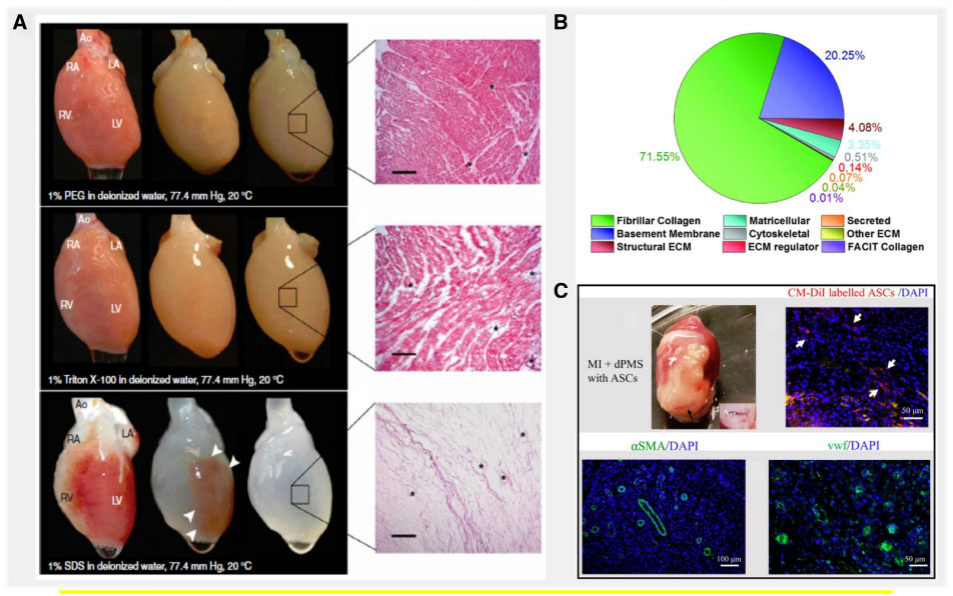
Advantages of using cardiac tissue-derived decellularized extracellular matrix for myocardial repair. (**A**) Perfusion decellularization of rat WHs using poly ethylene glycol (PEG), Triton X-100 and SDS. Corresponding H&E staining of dECM showed complete decellularization of rat heart perfused with SDS and incomplete decellularization in PEG and Triton X-100-treated hearts. Scale bar. 200 µm. Source: Adapted with permission from Ott *et al.* [14]. (**B**) Measurement of total proteins obtained from injectable human cardiac dECM using ECM-targeted quantitative conCATamers (QconCAT) by liquid chromatography–selected reaction monitoring (LC-SRM). Source: Adapted with permission from Johnson and Hill *et al.* [37]. (**C**) *In vivo* assessment of cardiac dECM generated by seeding adipose-derived stem cells (ASCs) in a rat myocardial infarction (MI) model. Vessel formation was demonstrated as shown by immunofluorescence staining with vascular markers α-SMA and vWF. Source: Adapted with permission from Shah *et al.* through open access policy [38]

Decellularized extracellular matrix (dECM) achieved from myocardium tissues have been widely used in tissue engineering and regenerative medicine because of the many benefits that cardiac dECM offers to develop strategies for myocardial repair (Fig. 2). After decellularization, the cardiac dECM scaffold has shown to provide a complex combination of biochemical and mechanical cues retained from native myocardium tissue that favors the cell attachment, proliferation and cardiovascular differentiation during subsequent recellularization [55, 56]. Recently, heart tissues obtained from multiple species (e.g. rat, pig and human) have been used to produce 3D dECM slices or acellular WH for biomedical applications, which has resulted the cardiac-specific functionality *in vitro* as well as *in vivo* after transplantation [34, 57, 58]. The importance of dECM bioactivity as therapeutics has been highlighted in many studies [15, 53]. Comparatively, very little is known about the contributions from dECM structure and composition. Several of recent studies indicated the important role of cardiac ECM composition on stem cell differentiation and cardiac development [59, 60]. Mechanical properties of biomaterial scaffolds have also been gradually recognized in determining the efficacy in tissue repair [61, 62]. Therefore, in addition to the witnessed importance of dECM bioactivity in cardiac repair, we believe that future studies that aim to investigate dECM composition and mechanical properties will help complement our understanding of cardiac dECM therapeutic mechanisms and move the clinical applications forward. In this review, we focused to provide an overview of recent progresses and emerging challenges using cardiac dECM scaffolds. The recent studies that have reported the use of cardiac dECM scaffolds for heart infarction treatment are summarized in Tables 1 and 2.

**Table 1.** Summary of cardiac tissue-derived dECM scaffolds used for producing cardiac patch for myocardial infarction treatment

Species	Decellularization agents	Scaffold thickness	Cell types	*In vitro*	*In vivo*	Biological results	Refs
Rat	1% SDS; 1% Triton X-100/0.5% EDTA	N/A	Human induced pluripotent stem cell-derived CMs; human induced pluripotent stem cell-derived CD90^+^ cells	×	×	Cardiac dECM scaffold enhanced the maturation of human iPSC-derived cardiac cells *in vitro*. After seeding, the dECM scaffold exhibited normal electrical properties and responded to the pharmaceutical agents. When patched on the acute rat MI model, the recellularized cardiac dECM has shown to reduce infract size, increase in wall thickness and promote vascularization	[26]
Rat	10 mM Tris HCl/0.1% EDTA; 0.2% SDS/10 mM Tris HCl	N/A	Immortalized adult Lin^-^Sca-1^+^ cardiac progenitor cells (iCPC^Sca-1^); neonatal rat CMs	×		Fetal and adult cardiac dECM scaffolds, when seeded with cardiac progenitor cells and neonatal CMs, have shown to support the viability, proliferation and migration. Compared to adult cardiac dECM, fetal scaffold has resulted better repopulation efficiency, migration and colonization rates of seeded Lin^-^Sca-1^+^ cardiac progenitor cells and neonatal rat CMs	[39]
Rat	0.25% Triton X-100/10 mmol/l NH_4_OH	10 µm*	Neonatal rat CMs	×		Neonatal rat CMs, when cultured on thin cardiac dECM slices, have exhibited higher proliferation rate and increased cardiac gene and protein expressions compared to the control group	[40]
Rat	1% SDS; 1% Triton X-100	N/A	Induced pluripotent stem cells	×		Rat cardiac dECM supported the attachment, survival, growth and differentiation of iPSCs as indicated by decreased pluripotency markers after 7 days of culture	[41]
Rat	1% SDS; 1% Triton X-100	381±157 µm	N/A	×		The rat cardiac dECM had significantly higher stiffness compared to the native myocardium tissue	[42]
Mouse	0.25% SDS/0.5 mg/ml DNase	N/A	Mouse embryonic ventricular cells; mouse ESC-derived progenitors	×		The recellularization of embryonic cardiac dECM with mouse embryonic ventricular cells and mouse ESC-derived progenitors (day 5 after inducing differentiation) has resulted ESC differentiation as characterized by endothelial, cardiac and smooth muscle markers, leading to achieve beating cardiac patch after 20 h and 24 days of culture, respectively	[27]
Mouse	0.05% Trypsin/0.02% EDTA, 1.1% NaCl and 0.7% NaCl; 0.1% SDS; 1% Triton X-100	300 µm	Murine ESCs	×		Cardiac dECM scaffold, when seeded with murine ESCs using hanging drop method, has shown to form cell aggregates which attached, survived, proliferated and merged with adjacent aggregates during 16 days of culture	[31]
Porcine and rat	SDS; Triton X-100	300 µm*	Neonatal rat ventricular cells	×		Neonatal rat ventricular cells, when seeded on rat or pig engineered heart slices, have promoted cell elongation, alignment and synchronous contraction, leading to the production of an anisotropic and functional tissue that could be electrically paced for electrophysiological studies	[43]
Porcine	1% Triton X-100, 1% SDS and 0.5% Trypsin	2000 µm*	Rat myocardial fibroblast; rat neonatal CMs	×		Compared to decellularization with Trypsin and Triton X-100, the SDS-based treatment has resulted better decellularization efficiency of porcine myocardium tissue as demonstrated by complete removal of cells and better preservation of ECM microstructures. When seeded on obtained dECM scaffolds, rMFs showed distinct cell attachment and growth rate response while rCMs were different in terms of morphologies and spontaneous beating magnitudes based on the decellularization methods.	[44]
Porcine	1.1% NaCl/0.02% EDTA and 0.7% NaCl/0.02% EDTA; 0.05% Trypsin/0.02% EDTA; 1% Triton X-100 and 0.1% ammonium hydroxide	1500 µm*	N/A		×	Decellularized porcine myocardium patch, when implanted on acute and chronic rat MI models, has shown to promote robust vascularization after implantation, recruit cardiac progenitor (GATA4^+^, c-kit^+^) and myocyte (MYLC^+^, TRPI^+^) on the patch and induce constructive ECM remodeling as indicated by increased M2/M1 macrophage phenotypic ratio, leading to the significant improvement of cardiac function	[28]
Porcine	0.1% SDS/0.01% Trypsin, 1 mM phenylmethylsulfonylfluoride and 20 µg/ml RNase A/0.2 mg/ml DNase	2000 µm*	Porcine bone marrow mononuclear cells	×		Porcine cardiac dECM, when cultured with the mixture of undifferentiated and differentiated bone marrow mononuclear cells toward cardiac phenotype, has supported cell attachment, viability, infiltration and proliferation of seeded cells, leading to maintain the CM-like phenotype and possible endothelialization within the scaffold.	[45]
Porcine	0.1% SDS/0.01% Trypsin, 1 mM phenylmethylsulfonylfluoride and 20 µg/ml RNase A/0.2 mg/ml DNase	2270 ± 380 µm	N/A	×		Porcine myocardium tissue, when treated with 0.1% SDS and 0.01% trypsin solution using a frame-pin supporting system and a rotating bioreactor, has resulted removal of cells, DNA fragments (∼98%) and α-Gal porcine antigens. Compared to native porcine myocardium tissue, dECM scaffold showed stiffer tensile properties	[46]
Porcine	0.1% SDS/0.01% Trypsin, 1 mM phenylmethylsulfonylfluoride and 20 µg/ml RNase A/0.2 mg/ml DNase	3000 µm*	Rat MSCs	×		Rat MSCs seeded on porcine dECM scaffold with a needle injection, when cultured using the CM differentiation growth medium containing 5-azacytidine and subjected to mechanical and electrical stimulations (20% strain; 5 V, 1 Hz), resulted in the differentiation of MSCs toward CM-like phenotype	[47]
Porcine	10 mM Tris/0.1% EDTA; 0.5% SDS; DMEM containing 10% fetal bovine serum; 0.1% peracetic acid/4% ethanol	150 µm*	Neonatal rat ventricular myocytes; human ESC-derived CMs; human induced pluripotent stem cell-derived CMs	×		When seeded with NRVMs, the laser-cut thin sheet of decellularized cardiac slices resulted synchronously beating and exhibited a striated pattern of organized sarcomeres. Decellularized cardiac ECM slices seeded with hESC-CMs produced beating scaffolds with measurable intracellular calcium transients and maximum twitch stress of 1.7 N/mm^2^. Similarly, hiPSC-CM seeded dECM slices achieved maximum peak stress of 6.5 mN/mm^2^ and twitch kinetics similar to the reported values from adult human trabeculae	[48]
Porcine	1.1% NaCl and 0.7% NaCl; 0.05% Trypsin/0.02% EDTA; 1% Triton X-100/1% ammonium hydroxide	14 600 ± 19 00 µm	Rat MSCs; human umbilical vein endothelial cells	×		When compared the decellularization protocols, Triton X-100/trypsin-based perfusion method was found to be more effective to achieve thicker dECM while retaining structural characteristics, inherent vasculature, fiber morphology and mechanical properties. The thick dECM scaffold supported the attachment and long-term cell survival of rMSCs. HUVECs were found to form a monolayer surrounding the inner lumen of the inherent vasculature on dECM	[30]
Porcine	1.1% NaCl and 0.7% NaCl; 0.05% Trypsin/0.02% EDTA; 1% Triton X-100/1% ammonium hydroxide	15 000 µm*	Bone marrow-derived MSCs; human umbilical vein endothelial cells; human ESC-derived CMs	×		Thick porcine myocardium dECM, when co-cultured with hMSCs and HUVECs under dynamic culture conditions using a perfusion bioreactor chamber, has supported compartmentalized recellularization and higher cell infiltration compared to static culture conditions, leading to functional vascularization/angiogenesis as indicated by sprouting of capillary-like vessels within the areas of scaffold containing high hMSCs (up to 1.7 mm thickness). Human ESC-derived CMs were seeded on thick dECM scaffold, and the scaffold supported CM phenotype, leading to synchronous beating 3 days after initial cell seeding	[49]
Porcine	1.1% NaCl/0.02% EDTA and 0.7% NaCl/0.02% EDTA; 0.05% Trypsin/0.02% EDTA; 1% Triton X-100 and 0.1% ammonium hydroxide	3000 µm*	Sheep cardiac fibroblast; rat cardiac myocytes; rat bone marrow-derived MSCs	×		Cardiac fibroblast seeded on porcine dECM resulted the scaffold shrinkage and ECM remodeling, as demonstrated by significant increase of GAG content (∼23%) and ECM remodeling-related mRNAs including collagen I and III, matrix metalloproteinase 2 and type 1 tissue inhibitor of metalloproteinase as compared to control. Decellularized ECM scaffold seeded with CMs began to beat few days after initial seeding and showed positive expression for functional cardiac markers. dECM seeded with MSCs maintained cell viability over 24 days in culture	[50]
Porcine	0.02% Trypsin/0.05% EDTA/0.05% NaN_3_; 3% Triton X-100/0.05% EDTA/0.05% NaN_3_; 4% deoxycholic acid	N/A	Chicken embryonic CMs	×		Perfusion-based decellularization of porcine WH resulted cardiac dECM with well-preserved collagen, elastin, and GAGs, and mechanical integrity. Cardiac dECM sheet supported the formation of organized chicken CM sarcomere structure *in vitro*, as indicated by α-actinin staining for striations fibers	[25]
Porcine	0.02% Trypsin/0.05% EDTA/0.05% NaN_3_; 3% Triton X-100/0.05% EDTA/0.05% NaN_3_; 4% deoxycholic acid	2500 µm	N/A		×	Porcine cardiac dECM patch, when used to treat right ventricular outflow tract (RVOT) defect in a Lewis rat model, has resulted CM recruitment, dECM patch remodeling and neovascularization, leading to the improved heart function	[51]
Porcine	1% SDS; 0.01% Triton X-100	300, 600 and 900 µm	hMSCs; rASCs	×		When cultured on top of the scaffold from one side (lateral cell seeding), decellularized porcine myocardial slices (dPMSs) supported the cell attachment with high viability and induced endothelial differentiation and maturation of hMSCs and rASCs. Compared to lateral seeding, bilateral cell seeding (cells were seeded from both sides of the scaffold) has significantly enhanced seeding efficiency, infiltration and growth in 600 μm dPMS	[52]
Porcine	1% SDS; 0.01% Triton X-100	300 µm	rASCs; pig adipose-derived stem cells	×	×	Rat and pig ASCs, when seeded on 300 μm dPMS, has shown the distinct responses in terms of cell attachment, viability, infiltration and proliferation. The rASCs cultured on dPMS showed endothelial differentiation. When used to deliver rASCs using dPMS on a rat MI model, a higher number of transplanted cells were present in the infracted area compared to direct injection seeding method, leading to increased vascular formation within the patch	[38]
Human and porcine	10 mM Tris/0.1% EDTA; 0.5% SDS	300 µm*	Human umbilical cord blood-derived MSCs; murine iPSC-derived CMs; murine neonatal CMs	×		Cardiac dECM scaffold promoted cell attachment, viability and proliferation of human umbilical cord blood-derived MSCs, murine iPSC-derived CMs and murine neonatal CMs. Compared to MSCs, iPSC-derived CMs showed less cell attachment, proliferation and infiltration on the human dECM slices	[29]
Human	1% SDS	N/A	Human cardiac progenitor cells; hMSCs; human umbilical-vein endothelial cells; H9c2 rat CMs; HL-1 CMs	×		When seeded on human dECM scaffold, hCPCs has shown to enhance the expression of cardiac markers including bMHC, MEF2C, Nkx2.5 and TnnT. HUVECs cultured on dECM formed a lining of endocardium and vasculature. The CMs organized into nascent muscles bundles and showed mature calcium dynamics and electrical coupling on dECM scaffold	[24]
Human	1% SDS; 1% Triton X-100	200 µm	Human iPSC-derived CMs	×		Human iPSC-derived CMs, when seeded on human dECM slices, has supported the cell attachment, viability and function as evidenced by spontaneously contracting slices within 4–7 days of culture and the presence of sarcomeric structure, cell-mediated matrix deformation, electrical conduction and contractile force	[36]
Human	10 mM Tris/0.1% EDTA; 0.5% SDS	300 µm*	Murine ESCs; murine induced pluripotent stem cells; murine mesenchymal stromal cells	×		Human cardiac tissue-derived dECM favored attachment, viability, proliferation, and cardiac lineage commitment of seeded ESCs and iPSCs as evidenced by positive immunohistochemistry staining for cardiac troponin T and heavy-chain cardiac myosin as well as significant increase of mRNA expression for myosin heavy polypeptide 6, cardiac troponin T2 and NK2 homeobox 5. MSCs showed no evidence of CM differentiation	[32]
Human	10 mM Tris/0.1% EDTA; 0.5% SDS 1% SDS; 1% Triton X-100 (3) 1% SDS and 1% Triton X-100	350 µm*	Human cardiac primitive cells	×		Human cardiac primitive cells, when cultured on dECM scaffold, has promoted the differentiation of seeded cells toward CMs and smooth muscle cells as indicated by distinct gene expression for CMs (MEF2C, ACTC1) and smooth muscle cells (GATA6, ACTA2)	[53]
Human	1% SDS	400 µm	Human ESC-derived CM-like cells; human induced pluripotent stem cell-derived CM-like cells	×		Human cardiac dECM scaffold, when cultured with hPSC-derived CLCs, has shown to promote the differentiation and maturation toward CMs as demonstrated by enhanced electrophysiological properties and positive immunofluorescence staining for alpha-sarcomeric actinin, Troponin T, MYH6, NKX2.5 and CX43 after 10 days of culture	[54]

The asterisk sign (*) denotes the thickness of dECM before tissue decellularization. For these studies, the scaffold thickness after decellularization procedure has not been reported. N/A indicates not applicable.

**Table 2.** Chemical modification of cardiac dECM scaffold for biomimetic cardiac patch

Species	Decellularization agents	Scaffold thickness	Cell types	Chemical modification method	*In vitro*	*In vivo*	Biological results	Refs
Porcine	1.1% NaCl and 0.7% NaCl; 0.05% Trypsin/0.02% EDTA; 1% Triton X-100/1% ammonium hydroxide	10 000 µm*	hMSCs; human umbilical vein endothelial cells	Decellularized ECM scaffold was coated with either 4 mg/ml gelatin or 10 µg/ml fibronectin or 100 µg/ml laminin by immersing the scaffold in the coating solution for 24 h	×		Chemically modified porcine cardiac tissue-derived dECM, when co-cultured with HUVECs and MSCs, has shown to support the cell growth and alignment on the surface and vasculature of reseeded scaffold	[100, 101]
Porcine	(1) 1% SDS; 1% Triton X-100; 0.1 mg/ml DNase I(2) 1.1% NaCl and 0.7% NaCl; 0.05% Trypsin/0.02% EDTA; 1% Triton X-100/1% ammonium hydroxide	3000 µm*	Adipose tissue-derived progenitor cells; porcine adipose tissue MSCs	Decellularized ECM was prepared by rehydrating the scaffold with RAD16-I peptide hydrogel followed by cell seeding using the suspension containing 10% sucrose solution.	×	×	Porcine dECM scaffold, when seeded with adipose tissue-derived progenitor cells, has shown to express endothelial marker isolectin B4 as well as cardiac markers including GATA4, connexin43 and cardiac troponin T. The cardiac dECM seeded with ATDPCs and ATMSCs, when implanted in a porcine MI model, has promoted neovascularization of the ischemic myocardium and resulted significant restoration of ventricular cardiac function	[102–104]
Human	10 mM Tris/0.1% EDTA; 0.5% SDS; 50 U/ml DNase and 1 U/ml RNase	300 ± 50 µm	Human mesenchymal progenitor cells	Composite scaffold was produced by seeding TGF-β-treated MPCs on dECM in a PBS solution containing 2% fibrinogen and 100 U/ml thrombin, followed by adding growth medium supplemented with 0.1 ng/ml of TGF-β		×	When used to treat the injured heart using a rat MI model, the composite scaffold promoted the formation of vascular networks in the infract bed, leading to functional recovery of left ventricular systolic dimensions and contractile properties as evidenced by echocardiography analysis	[105]


The asterisk sign (*) denotes the thickness of dECM before tissue decellularization. For these studies, the scaffold thickness after decellularization procedure has not been reported.

### Preserved organ-specific structure and physical properties

Cardiac tissue-derived dECM offers many unique advantages for biomedical applications due to preservation of organ-specific microstructure, vasculature, mechanical integrity and scaffold degradability that promotes cell attachment, growth and cell–ECM interaction [63–65]. Several studies have reported that, after proper decellularization, cardiac dECM could retain intact geometry and vasculature tree of native heart which makes it suitable nature platform for fabricating engineered construct for cardiac repair [66–69]. For example, by coronary perfusion with detergent solution containing 1% SDS and 1% Triton X-100, Ott *et al.* reported the first decellularization of a rat WH into an acellular ECM with perfusable vascular architecture, competent acellular valves and four-chamber geometry. The obtained acellular rat WH also showed the retention of both larger cardiac vessels and smaller third-level and fourth-level branches. The left main coronary artery and the aortic root architecture were preserved within the decellularized WH. In addition, the equibiaxial mechanical testing demonstrated the retained mechanical properties after decellularization as indicated by anisotropic stress–strain behavior, high tangential modulus and similar membrane stiffness compared to native heart [14]. Similarly, Wang *et al.* reported the retention of vasculature and ultrastructure after decellularization of porcine myocardium tissue using a frame-pin supporting system in a rotating bioreactor containing 0.1% SDS and 0.01% trypsin solution [45–47]. Masson’s trichrome staining and SEM images were used to show the removal of cells and preservation of the interconnected 3D cardiomyocytes lacunae. The presence of cardiac elastin ultrastructure and vascular elastin distribution/alignment within the porcine myocardium dECM was demonstrated by Movat’s pentachrome staining. Compared to native myocardium tissue, both uniaxial and biaxial mechanical testing along fiber-preferred direction and cross-preferred direction showed a stiffer mechanical response of dECM scaffold as confirmed by stress–strain curve, which were found to be recovered after recellularization due to increased cellular content [45–47]. These reports have shown the potential of decellularization for removing cellular components from the native myocardium tissue while maintaining the mechanical properties and vascular networks critical for subsequent recellularization. It also needs to be noted that dECM are degradable and serves as a temporary scaffold when implanted as a cardiac patch. The preservation of structural and mechanical characteristics of dECM after implantation could bring many benefits for assisting cardiac repair and regeneration. First, dECM could provide the injured myocardium with effective mechanical compensation. Tissue mechanical properties are mainly determined by ECM. Therefore, cardiac dECM, when applied as a cardiac patch, could withstand the continuous contraction/relaxation of the heart and mechanically stabilize the infarcted region to prevent or slow down the negative remodeling process. Animal studies have shown that the initial dECM degradation after implantation could lead to the decreased scaffold mechanical properties [70, 71]. However, once the infiltrated cells start producing new ECM, the scaffold undergo rapid remodeling that helps recovering the mechanical strength [65]. Second, the structural and mechanical characteristics of dECM could serve as physical cues to the delivered stem cells or infiltrated host progenitor cells to facilitate cardiovascular differentiation and heart regeneration. Last, the degradation of dECM would eliminate any possible long-term side effects associated with dECM implantation such as chronic inflammation.

### Retained cardiac ECM chemical components and composition

Decellularization of cardiac tissue has shown to maintain major cardiac ECM components and composition which contains unique combination of chemical and biological cues for mimicking native microenvironment. Such preservation of biochemical cues within dECM could be beneficial for cell attachment, growth and stem cell differentiation as demonstrated in many previous studies [72–74]. For example, Johnson and Hill *et al.* performed a thorough study to confirm the retention of ECM proteins after decellularization using human myocardium tissue via proteomic approaches. Decellularized human myocardium tissue, when analysed by polyacrylamide gel electrophoresis (PAGE) and liquid chromatography tandem mass spectroscopy (LC-MS/MS), has shown to consist over 200 distinct cardiac ECM proteins including collagen, laminin, elastin and glycosaminoglycans (GAGs) [37]. Similarly, our group has also examined and confirmed the presence of complex mixture of ECM components (e.g. collagen and GAGs) within the decellularized tissue from porcine myocardium [75]. In addition, several studies have reported that cardiac decellularized ECM also retained the soluble matrix-bound growth factors after decellularization process [15, 53]. For instance, Methe *et al.* demonstrated the presence of various matrix-bound growth factors and cytokines within the decellularized porcine myocardium tissue that involved in angiogenesis (e.g. vascular endothelial growth factor family and fibroblast growth factor), cardiac homeostasis and remodeling (e.g. leptin, endothelin and angiotensin), and mitogenic cardiokines (e.g. hepatocyte growth factor, endoglin and bone morphogenetic protein-9). Furthermore, the authors also found several proteins that induce proliferation, survival, differentiation and recruitment of cells in response to inflammation (e.g. granulocyte colony-stimulating factor and interleukin-8) in the decellularized porcine myocardium tissue [15]. The cumulative results of these studies have confirmed the preservation of tissue-specific ECM components and composition within cardiac dECM after decellularization, which has significant role to maintain biomechanical properties of scaffold, promote cell–matrix interaction and modulate cell behavior during recellularization [76–78]. In terms of above reports, the component types and amounts in dECM are much more important than their ultrastructure for dECM patches as well as injectable ECM hydrogel. Thus, developing a mild decellularization technique to maximally maintain bioactive components and completely removing cells and immune agents are the keys to achieve excellent dECM materials.

### Served as cardiac microenvironment for cells

The development of effective decellularization strategies has greatly facilitated the use of cardiac dECM as an *in vitro* platform by providing biomimetic microenvironment which has shown to influence the cell behavior [79–81]. Increasing evidence has demonstrated that cardiac dECM could be used to direct robust cardiovascular differentiation and maturation of stem cells when used as a substrate for *in vitro* and *in vivo* studies [26, 40, 54]. Because of these advantages, cardiac dECM derived from multiple species (e.g. rat, porcine and human) have been explored to investigate the recellularization and differentiation potential by reseeding the scaffold with various stem cells. For example, the thin slice of decellularized human myocardium tissue (300 µm thick), when reseeded with murine ESCs or murine iPSCs or murine mesenchymal stromal cells, has shown to support cell attachment, viability, proliferation and CM differentiation of ESCs and iPSCs as indicated by significant increase of mRNA expressions for cardiac alpha myosin heavy chain 6, cardiac Troponin T2 and NK2 homeobox 5 (Nkx 2.5) as well as positive immunohistochemistry staining for cardiac troponin T and cardiac myosin heavy chain. Murine mesenchymal stromal cells showed no evidence of differentiation toward cardiac lineage [32]. Similarly, by injecting rat MSCs on decellularized porcine myocardial tissue (3000 µm thick), Wang *et al.* reported the differentiation of MSCs into CM-like phenotype using differentiation induction medium containing 5-azacytidine, as revealed by positive expression of sarcomeric α-actinin, myosin heavy chain, cardiac troponin T, connexin-43 and N-cadherin. When applied combined mechanical and electrical stimulations (20% strain; 5 V and 1 Hz) simultaneously using a multistimulation bioreactor system, the seeded cells demonstrated enhanced CM differentiation, high cell density and tissue remodeling within the scaffold compared to the static control group [47]. In our previous study, we also demonstrated the accelerated vascular differentiation of human mesenchymal stem cells (hMSCs) and rat adipose-derived stem cells (rASCs) when cultured on dECM slices as evidenced by positive immunofluorescence staining for early and late endothelial cell markers including CD31, von Willebrand factor, VE cadherin and alpha smooth muscle actin [52]. In the recent years, dECM tissue has also been used to facilitate maturation of differentiated stem cells to engineer functional cardiac patches for therapeutic outcomes. For instance, mouse ESC-derived progenitor cells, when used to reseed on dECM scaffold obtained from mouse embryonic myocardium tissue, has resulted to promote cardiac differentiation and maturation as evidenced by low expressions of stage-specific embryonic antigen-1, smooth muscle actin and CD31 markers and high expression of α-actinin, leading to produce beating cardiac tissue construct within 20 days of culture [27]. Similarly, Wang *et al.* generated functional cardiac patches using rat decellularized myocardium tissue and human iPSC-derived cardiac cells which exhibited normal contractile and electrophysiological properties *in vitro*. When used to patch on the rat heart with acute MI, the recellularized patches improved heart function as indicated by reduced infract size and recovery of left-ventricular ejection fraction, compared to acellular ECM patch group or MI control group [26]. In addition to cardiovascular stem cell differentiation and maturation, cardiac dECM have successfully exhibited its potential to recruit stem or progenitor cells from endogenous sources through several mechanisms including the release of matrix-bound growth factors and pro-angiogenic factors [28, 82, 83]. These findings have demonstrated the feasibility of cardiac dECM scaffold for providing suitable microenvironment during recellularization, stem cell differentiation and maturation, and cell recruitment toward the fabrication of functional patch for cardiac repair.

## Challenges of cardiac decellularized extracellular matrix and future directions

The optimal decellularization of cardiac tissue requires the complete removal of cellular components without affecting the structural characteristics, biochemical cues and mechanical integrity of the 3D ECM [65, 84]. Many groups have explored various decellularization and recellularization strategies that aim to obtain biomimetic myocardium tissue using cardiac dECM scaffold and have shown great promises in myocardial repair. However, the dECM derived from myocardium tissue still possesses many challenges for completely recapitulating the native heart. Some of the major issues that need to be addressed include the variations caused by different decellularization methods, insufficient preservation of vasculature and ECM composition, inhomogeneous recellularization within the scaffold compartments and diffusion limitation of thick cardiac dECM [6, 34, 84].

### Balance between cell removal and extracellular matrix preservation

Various approaches have been explored to investigate the optimal decellularization parameters for cardiac tissue including the use of different decellularization methods (e.g. perfusion and agitation), agents (e.g. chemical treatments, biological treatments and physical treatments) and treatment duration [56, 85, 86]. The common decellularization methods that have been widely used for the cardiac tissue are perfusion and agitation [17, 50, 53, 87]. Perfusion employs the continuous infusion of the decellularization solution through the vasculature of heart using a bioreactor system. This technique could greatly facilitate homogeneous exposure of the decellularization perfusates across the WH for removing the cellular components. However, the perfusion pressure and force used during the decellularization process could damage ECM and affect mechanical properties of the obtained dECM [81]. Agitation utilizes immersion of cardiac tissue into the decellularization solution with gentle mechanical shaking. It can be used for decellularizing small tissue sections when the access to the vasculature is difficult. However, compared to perfusion method, it requires longer exposure time to the decellularization agents that could disrupt ECM due to excessive treatment and agitation [81]. Similarly, the chemical detergent such as SDS, when used for tissue decellularization, has shown to effectively remove cellular components but also damaged collagen, GAG content and growth factors leading to disrupt ECM ultrastructure [88–90]. The prolonged use of trypsin (biological enzyme) for decellularizing heart tissues has demonstrated to reduce major ECM components (e.g. collagen, laminin, fibronectin, elastin and GAG content) after the decellularization procedure [5]. In addition, the exposure duration of tissue to the decellularizing solution also plays a key role for maintaining the structural and functional proteins. For instance, when used the shorter treatment time, the obtained dECM has shown the incomplete removal of cell debris but better retention of ECM components. However, the dECM has shown to suffer low maintenance of ECM proteins and mechanical strength when decellularized using the longer treatment time [5, 6, 56]. Therefore, the current decellularization strategies require further optimization for the experiment parameters by finding the appropriate complexity and duration of the decellularization treatments to produce the optimal cardiac dECM.

The therapeutic outcomes of using dECM in cardiac repair are largely determined by the quality of the dECM. The balance between cell removal and preservation of cardiac ECM properties (e.g. structural, biochemical and biomechanical cues) is crucial to achieve an optimal dECM scaffold that will minimize the immunogenicity upon implantation and achieve desired cell-ECM interaction. *In vivo* studies have proved that the remnant genetic materials (e.g. DNA, RNA and antigens) within dECM could trigger an inflammatory response leading to immune-mediated rejection after implantation [91, 92]. For example, Brown et al. evaluated the host response of cellular porcine bladder tissue and acellular porcine bladder ECM using a rat abdominal wall defect model. When implanted the porcine bladder tissue containing xenogeneic cells in rats, the scaffold showed a classic cascade of inflammatory reaction as indicated by presence of mononuclear cells including macrophages predominately of M1 phenotype, leading to scar tissue formation. In contrast, the acellular porcine bladder ECM resulted the shift in macrophage phenotype from M1 to M2 that promoted constructive tissue remodeling [93]. To reduce immunogenicity in decellularized tissues, Crapo et al. recommended the minimum criteria for effective removal of cellular materials from dECM by maintaining <50 ng dsDNA per mg dry weight of dECM, <200 bp DNA fragment length, and lack of nuclei in tissue sections stained with 4’, 6-diamidino-2-phenylindole (DAPI) and hemotoxylin and eosin (H&E) [5]. However, it remains a hurdle to completely remove the cellular materials and maintain the intact 3D ECM integrity using the current decellularization strategies. Increasing evidence has suggested that the treatment methods and agents could alter the structure and composition of ECM and affect the decellularization efficiency [5, 94]. For example, Akhyari et al. performed a direct comparison by decellularizing the rat WH via perfusion using four decellularization protocols consisting of different treatment agents, concentration, and duration. When compared the decellularization efficiency of decellularized whole heart (dWH), the tested protocols have shown to produce distinct dECM scaffold in terms of cell removal, ECM microstructure and biochemical conservation. Moreover, the decellularization procedures that resulted better preservation of ECM proteins (e.g. laminin, elastin, and GAGs) could not completely remove residual DNA from the tissue. Conversely, when significant reduction of cell debris was achieved, the dECM scaffold suffered from low retention of ECM proteins [95]. Similarly, by decellularizing human cardiac tissue (350 µm thick) using five different decellularization methods in an orbital shaker with gentle agitation, Meglio *et al.* generated human dECM scaffold that differed in architecture, composition, and ability to support engraftment, survival, and differentiation of cardiac primitive cells [53]. In addition to genetic materials, the excess remaining of residual detergents and endotoxins in decellularized tissues and change in ECM fiber structure within the scaffold could also contribute to induce inflammation [2]. These findings have proved the variations of decellularization efficiency within dECM based on the decellularization strategies which could have potential impact for their downstream applications.

In the future, the protocol could be tailored specific for cardiac tissue to obtain an ideal dECM by minimizing the undesirable effects associated with the decellularization procedure. Specifically, the decellularization protocol should be standardized for cardiac tissue by (i) optimizing the parameters such as concentration for decellularizing agents, decellularization mode (e.g. agitation vs. perfusion), exposure time and the right combination of decellularization strategies (e.g. chemical, biological and mechanical treatments) and (ii) establishing the common criteria to evaluate the effectiveness of decellularization including removal of cells and DNA content, quantification of residual detergents, preservation of ECM components, and biomechanical properties and maintenance of 3D architecture and vascular integrity.

### Recellularization of cardiac decellularized extracellular matrix

The full recellularization is required to enable the construction of a functional cardiac tissue using dECM. The cardiac dECM scaffold must be recellularized by precise positioning of specific cell types (e.g. endothelial cells for vasculature network) to mimic the natural heart functions including contractility, electrical conduction and drug response [34, 81, 96]. Previous reports have demonstrated that the recellularized cardiac dECM could improve heart function when implanted in the acute and chronic MI animal models. For example, when patched on the acute rat MI model, the cardiac dECM generated by seeding a mixture of human iPSC-derived CMs and iPSC-derived CD90^+^ cells, has shown to reduce infract size, increase wall thickness and promote vascularization as indicated by positive staining of vWF and α-SMA, leading to improve the heart function compared to the control group [26]. Similarly, when used to deliver rASCs on a rat MI model, recellularized cardiac dECM resulted in the presence of higher number of transplanted cells at the infracted area compared to direct injection seeding method, leading to increased vascular formation within the patch [38]. These findings demonstrated the importance of recellularization for therapeutic benefits of cardiac dECM. However, efficient recellularization of cardiac dECM has not been achieved which has compromised the advantages of scaffold when used to treat the cardiovascular diseases. Some of the key limiting factors in the current recellularization strategies for cardiac dECM scaffold include inhomogeneous cell distribution, poor long-term cell survival and the need for right combination of multiple cell types for recapitulating the native heart function [97].

Many groups have explored various approaches to optimize the recellularization strategies for cardiac dECM including the use of different cell types and seeding methods [82, 98, 99]. The most commonly employed seeding methods for recellularization of cardiac dECM include static seeding, cell injection and perfusion seeding. The static cell seeding method employs the cell suspension to passively introduce on top of the cardiac dECM scaffold. This seeding strategy has shown to form a cell monolayer on surface of the dECM scaffold or exhibit low seeding efficiency when successful in penetrating the dECM scaffold. For instance, decellularized porcine myocardial slices (dPMSs) (300 µm thick), when seeded with neonatal rat ventricular cells (NRVCs) on top of the scaffold using static seeding method, has resulted inhomogeneous distribution of NRVCs as indicated by the presence of high cell density near the surface (∼3 µm below periphery) compared to the core of dECM (∼30 µm below periphery) [43]. When seeded on top of dPMSs using static seeding technique, our previous studies have also shown limited infiltration of hMSCs and rASCs on 300, 600 and 900 µm scaffolds [38, 52]. Cell injection seeding method involves seeding of cells with a small gauge needle by multiple injections throughout the different regions of dECM scaffold [47]. It can deliver correct type of cells at the specific site of dECM scaffold. However, cell injection seeding method can lead to inhomogeneous distribution of cells within the scaffold. Similarly, perfusion seeding method uses a bioreactor system to perfuse cell suspension back and forth within the dECM scaffold. It can distribute cells throughout the scaffold and transport necessary nutrients and oxygen to promote cell survival. However, the seeding of cells by perfusion could compromise the initial seeding efficiency. For example, decellularized porcine WH, when infused with human umbilical vein endothelial cells (HUVECs) into coronary arteries via aorta followed by seeding of neonatal rat CMs through five intramural injections, has led to incomplete recellularization of HUVECs due to loss of cells during perfusion and achieved 50% cellularity of CMs at the injection sites while less cells in the distal sites [22]. Therefore, efficient strategies must be developed to enable thorough recellularization and take full advantages of dECM for cardiac tissue engineering applications.

The development of optimal recellularization strategies may improve seeding efficiency, cell distribution and survival within the cardiac dECM. The effective seeding method and culture condition could potentially improve the cell retention and infiltration within the dECM scaffold. For instance, the recellularization of dWH was improved by multiple seeding routes and cell infusions using perfusion-based seeding of cells through the vascular tree followed by continuing culture in a bioreactor system [83]. Similarly, our previous study has demonstrated that the bilateral cell seeding method (cells seeded from both sides of scaffold) could be used to achieve uniform cell distribution within 600 µm thick decellularized porcine myocardial slice [52]. Also, the cardiac dECM scaffold could be functionalized with different pro-angiogenic factors and ECM proteins to enhance initial cell attachment and vasculogenesis [100–105]. The cardiac dECM could be combined with cell sheet during the recellularization process to improve cell seeding efficiency and culture using a perfusion-based bioreactor culture system to promote cell infiltration. In addition, the bioreactor culture system could be designed for providing electrical and mechanical stimulations to mimic *in vivo* conditions and facilitate *in vitro* stem cell differentiation and maturation. On the other hand, through pepsin digestion and neutralization, the dECM has been processed into an injectable hydrogel, which has been directly delivered into the infracted heart to restore heart function [37, 72–74, 76]. The cells can be easily mixed with the pre-hydrogel solution, and then form a recellularized construct [106, 107].

### Tailoring material properties of cardiac decellularized extracellular matrix

The extent of ECM preservation after decellularization influences the material properties (e.g. mechanical strength, bioactivity and scaffold degradation) of cardiac dECM scaffold. After decellularization of cardiac tissue, the maximum retention of ECM components and structure is beneficial to regulate cell behavior *in vitro* and tissue regeneration *in vivo* [108, 109]. Emerging studies have demonstrated that decellularization procedure alters ECM composition and affects mechanical properties of the obtained cardiac dECM. For example, compared with native myocardium tissue, the decellularized myocardium tissue showed stiffer mechanical response as indicated by biaxial and uniaxial mechanical testing and reduced angiogenic growth factors as evidenced by Luminex technology [15, 45, 46]. By tuning the scaffold properties, the cardiac dECM could improve the mechanical strength, bioactivity, and degradation kinetics and therefore better mimic native tissue microenvironment. Moreover, the ability to control the material properties of cardiac dECM could enhance cell–ECM interaction after reseeding and facilitate cell growth, migration and differentiation.

Decellularized ECM derived from myocardium tissue could be modified through various biochemical treatments such as growth factors and bioactive molecules to tune scaffold properties, optimize degradation rate, enhance the bioactivity and promote vascularization. Recently, a few groups have already begun to explore various strategies to demonstrate the feasibility of composite cardiac dECM through scaffold modification and have shown encouraging *in vivo* results after implantation (Table 2). For example, by treating human mesenchymal progenitor cells and human cardiac dECM with transforming growth factor beta (TGF-β), Godier-Furnémont *et al.* produced composite scaffold that promoted the formation of vascular networks when used to treat injured heart using a rat MI model, leading to functional recovery of left ventricular systolic dimensions and contractile properties [105]. Similarly, the porcine cardiac dECM, when prepared by rehydrating with RAD16-I peptide hydrogel followed by seeding of porcine adipose tissue MSCs in a suspension containing 10% sucrose solution, has resulted to restore ventricular cardiac function in a pig MI model by reducing infract size and promoting neovascularization within the ischemic myocardium [102–104]. It is rarely found to combine heart tissue-derived dECM with other polymer materials as a cardiac patch. Many groups have explored the feasibility of fabricating 3D scaffolds by combining other types of dECM with synthetic/natural polymers [110–113]. Recently, Pok *et al.* constructed cardiac scaffold by mixing powered porcine myocardial matrix solution with chitosan followed by lyophilization to form 3D scaffold. The obtained dECM-chitosan scaffold, when seeded with neonatal rat ventricular myocytes, has demonstrated to improve the contractile and electrophysiological functions [114]. In another study, by combining dECM obtained from bovine pericardium tissue with poly(propylene fumarate) (PPF), Bracaglia *et al.* developed PPF-pericardium hybrid that supported cellular infiltration with improved inflammatory response when tested in rat *in vivo* model [115]. Additionally, several studies have also examined composite hydrogels by combining the heart ECM hydrogel with synthetic or natural hydrogel for cardiac repair such as dECM/collagen hydrogel and dECM/polyethylene glycol (PEG) hydrogel, which has shown improved mechanical properties and degradation rate [107, 116]. These studies showed that the combination of dECM with other biomaterials would be a promising way to improve dECM properties for cardiac repair. Combining decellularized ECM and bioactive molecules to generate composite scaffold is relatively new technique in cardiac tissue engineering. There are still many questions that need to be thoroughly investigated using composite dECM scaffold such as the effect of composite scaffold on cell behavior, mechanical properties and degradation rates.

Recently, dECM derived from cardiac tissue has also been explored to be used for biofabrication technologies such as electrospinning and 3D bioprinting [117–125]. Cardiac constructs have been prepared by electrospinning of dECM in conjunction with synthetic polymers for cardiac repair applications. For example, Schoen *et al.* demonstrated the fabrication of electrospun scaffold using porcine cardiac tissue-derived dECM and poly(ethylene oxide) with well-defined microstructures that supported the growth and survival of hMSCs and neonatal CMs [126]. In addition, dECM has shown great potentials to serve as a nature bioink for 3D bioprinting. Using porcine myocardium tissue-derived dECM bioink, rat myoblast cells and polycaprolactone (PCL) framework, Pati *et al.* constructed cardiac tissue blocks with the 3D bioprinting technique that maintained high cell viability and myoblast cells maturation [122]. Photocrosslinkable dECM bioinks have been developed for fabricating patient-specific tissues with high control over complex microarchitecture and mechanical properties [119]. Cardiac patches have been 3D printed with bioinks composed of cardiac dECM, human progenitor cells, and gelatin methacrylate (GelMA) and tested *in vivo* for myocardium repair [120]. These studies have demonstrated the promises of applying dECM in biofabrication strategies. We envision in the future that the composite scaffold based on chemically modified dECM and biofabrication strategies could be used to address some of the current challenges associated with decellularization and recellularization of cardiac dECM. The ability to tune ECM properties can provide powerful platform to optimize the culture conditions for constructing prevascularized and functional 3D myocardium tissue. For instance, the prevascularization of cardiac dECM scaffold could be achieved through scaffold functionalization with pro-angiogenic factors (e.g. vascular endothelial growth factor and basic fibroblast growth factor) and appropriate cell sources (e.g. endothelial cells and MSCs). The composite scaffold could also be tailored to guide stem cell differentiation and maturation toward the fabrication of functional cardiac tissue.

## Conclusions and perspectives

dECM derived from myocardium tissue has gained significant attention for cardiac repair and regeneration. The use of decellularization and recellularization strategies have enabled to produce cardiac dECM scaffold that mimic tissue properties (e.g. ECM architecture, biochemical cues and mechanical integrity) similar to native heart which favors cell attachment, growth, infiltration and differentiation. Because of these attractive advantages, cardiac dECM scaffold has potential to provide microenvironment and biological signals for damaged heart that could promote tissue reconstruction upon implantation. However, there are several issues that need to be resolved prior to clinical applications of cardiac dECM which includes finding optimal decellularization method, preservation of vasculature and ECM composition, recellularization strategies to properly reintroduce cells into the specific compartment of the scaffold and prevascularization of thick cardiac dECM. The thorough optimization of decellularization parameters and recellularization techniques will lead to further improve the engineered patch for myocardial repair. Recently, dECM has been commercialized and tested in clinical trials for myocardium regeneration [68, 127]. However, most studies used dECM derived from noncardiac tissues such as CorMatrix that is made of decellularized SIS [128–132]. It was reported that heart ECM-derived hydrogel (Vetrigel, Ventrix, Inc.) has been used for post infarction treatment in clinical trial (Phase 1) (ClinicalTrials.gov Identifier: NCT02305602). However, limited clinical results have been reported using decellularized cardiac ECM patch. Compared to the SIS-derived dECM, cardiac dECM would be more promising for future clinical applications in treating cardiac diseases. However, scaling-up and batch-to-batch variations are the two major concerns for commercialization. Currently, there are no good approaches to avoid batch-to-batch variations caused by the individual differences. Furthermore, the safety and efficacy of the dECM as cardiac patch needs to be evaluated. The dECM patches were investigated in small rodent animal models, but very few studies have been conducted to test decellularized heart ECM patches in large animal models [28, 51]. In addition, long-term investigation after implantation still needs to be executed prior to commercialization and clinical trial. Nevertheless, it is undoubted that the decellularized heart ECM scaffolds will be attractive candidates for clinical trial once they can be further characterized in detail.

## Funding

This work was partially supported by the National Institutes of Health (1R15HL122949 to G.Z., 1R15HL140503 to Y.H.), and the American Heart Association (19AIREA34400087 to G.Z.).

*Conflict of interest statement*. None declared.
